# Endometriosis‐Associated Periodontal Disease: A Large Cohort Perspective Study

**DOI:** 10.1111/odi.70044

**Published:** 2025-07-26

**Authors:** Maria Teresa Agneta, Giuseppe D'Albis, Letizia Lorusso, Nicola Bartolomeo, Antonia Abbinante, Anna Antonacci, Pietro Signorile, Francesco D'Aiuto, Elisa Mazza, Massimo Corsalini, Saverio Capodiferro

**Affiliations:** ^1^ Italian Association of Dental Hygienists (AIDI) Bari Italy; ^2^ Interdisciplinary Department of Medicine Department of Dentistry, University of Bari Aldo Moro Bari Italy; ^3^ Interdisciplinary Department of Medicine School of Medical Statistics and Biometry, University of Bari Aldo Moro Bari Italy; ^4^ President of the Italian Endometriosis Foundation Rome Italy; ^5^ Periodontology Unit UCL Eastman Dental Institute London UK; ^6^ Department of Clinical and Experimental Medicina University Magna Græcia Catanzaro Italy; ^7^ Technical Scientific Association of Food Nutrition and Dietetics (ASAND) Catania Italy

**Keywords:** chronic inflammation, endometriosis, multidisciplinary care, oral‐systemic connection, periodontal disease

## Abstract

**Objective:**

Endometriosis is a chronic estrogen‐dependent gynecological disease affecting millions of women worldwide. Besides its well‐established impact on reproductive health, emerging evidence suggests a potential association between endometriosis and periodontal disease. The present study discusses the interplay between the two diseases, exploring shared immunopathological mechanisms, including chronic inflammation, hormonal imbalance, and immune system modulation.

**Materials and Methods:**

A 45‐question standardized questionnaire was answered by 4072 women clinically diagnosed with endometriosis to identify self‐reported periodontal status, oral hygiene habits, and systemic symptoms.

**Results:**

Findings report that more severe stages of endometriosis are related to increased awareness of periodontal disease, greater gingival bleeding, xerostomia, and mucosal alterations. Moreover, greater diagnostic latency aggravates both systemic and oral disorders, supporting the necessity of early treatment. These findings highlight the need for a single, multidisciplinary standard of patient care involving gynecologists, dentists, dental hygienists, and other healthcare providers.

**Conclusion:**

By connecting gynecological and dental care, this study aims to promote awareness, facilitate early diagnosis, and improve quality of life for women suffering from endometriosis. Future research must explore mechanisms linking the conditions and assess therapeutic approaches to alleviate oral health issues in these individuals.

## 1 Introduction

According to United Nations data, endometriosis affects an estimated 3 million women in Italy, 14 million in Europe, and 150 million globally (Somigliana et al. 2023).

Endometriosis is a chronic, estrogen‐dependent, and benign gynecological disease with a complex, multifactorial pathogenesis. It is characterized by the ectopic implantation and proliferation of functional endometrial tissue outside the uterine cavity. The precise pathophysiological mechanisms remain poorly understood; however, exposure to environmental estrogens is implicated as a significant risk factor, potentially triggering the pathological cascade in individuals with a genetic or epigenetic predisposition (Vannuccini et al. 2022).

Despite advances in medical research, diagnostic and therapeutic strategies for endometriosis remain inadequate. A definitive diagnosis still relies exclusively on surgical intervention, complicating efforts to estimate the true prevalence of the disease. Endometriosis affects approximately 10%–20% of women of reproductive age, spanning from adolescence to menopause, with peak incidence occurring between 30 and 40 years. This prevalence is independent of ethnicity and socioeconomic status. Notably, 20%–25% of cases remain asymptomatic and undiagnosed for years, whereas 75%–80% of affected individuals experience debilitating symptoms, including severe pelvic pain, heavy menstrual bleeding, intermenstrual spotting, dysuria, dyschezia, chronic lower back pain, gastrointestinal disturbances, dyspareunia, and infertility affecting 30%–40% of women with endometriosis (Zondervan et al. 2018).

Beyond the systemic burden of the disease, emerging evidence suggests a correlation between endometriosis and periodontal diseases, highlighting a potential link between systemic inflammatory disorders and oral health. This association may be driven by shared immunopathological mechanisms, including dysregulated cytokine profiles (such as elevated IL‐6, IL‐1β, and TNF‐α), chronic low‐grade inflammation, and impaired immune regulation (Koninckx et al. 2019).

From a microbiological perspective, both conditions have been associated with alterations in the local microbiome composition. In periodontitis, the overgrowth of pathogenic species such as *Porphyromonas gingivalis* , *Treponema denticola* , and *Tannerella forsythia* disrupts the microbial homeostasis of the gingival sulcus, contributing to tissue destruction and systemic inflammatory responses. Similarly, recent studies have identified microbial dysbiosis in the peritoneal fluid and reproductive tract of women with endometriosis, suggesting a possible role for microbial translocation and immune activation in disease progression (Amro et al. 2022; Garcia Garcia et al. 2023).

Moreover, hormonal therapies and immunomodulatory drugs used in the management of endometriosis may exacerbate oral conditions by altering salivary flow and mucosal immunity, thereby increasing the risk of xerostomia, oral candidiasis, and mucosal inflammation, and potentially aggravating periodontal tissue breakdown (Culley et al. 2013; Van Niekerk et al. 2019).

Based on emerging evidence of shared inflammatory and immunological pathways, it is hypothesized that women with endometriosis may exhibit a higher prevalence of periodontal diseases, potentially contributing to a reduced quality of life (Evans et al. 2019).

Oral health is increasingly recognized as an integral component of systemic well‐being. This study aimed to assess, through a structured cognitive questionnaire, potential associations between endometriosis and self‐reported periodontal diseases, evaluating their collective impact on quality of life.

## 2 Material and Methods

A designed 45‐item structured questionnaire was administered to a cohort of 4079 women clinically diagnosed with endometriosis. Participants were recruited via targeted online communities within the social networking platform Facebook, leveraging these specialized groups to access a representative sample. The questionnaire was developed in collaboration with a multidisciplinary team of healthcare professionals from the University of Bari Aldo Moro, including gynecologists of the Italian Endometriosis Foundation in Rome, as well as dentists, dental hygienists, physiotherapists, dieticians, and psychologists. The questionnaire was designed to investigate the following key domains: endometriosis status, types of treatments received, manifestations and symptoms within the oral cavity (including self‐reported oral health and periodontal conditions), oral hygiene practices, dietary habits, physical activity, quality of life, and the presence of comorbidities associated with immune function. The final section of the questionnaire included an open‐ended question, allowing participants to provide personal perspectives and experiences. Data collection was conducted between April and June 2024. The present study was carried out according to the Declaration of Helsinki, and the research protocol was approved and registered in the Local Ethical Committee of Calabria Region Central Area (code 355/2021/CE).

The first section of the survey focused on demographic information, endometriosis diagnosis, and the participants' medical history. The second section examined oral disorders potentially linked to both pharmacological and non‐pharmacological treatments, with specific questions addressing whether participants observed oral changes after medication use or experienced symptoms such as oral bleeding. The third section targeted health, wellness, and lifestyle factors. Each question was assigned a unique code (e.g., Q1, Q2, etc.) to streamline data collection and analysis. Open‐ended responses were excluded from the quantitative analysis (Table 1).

**TABLE 1:** Question list collected in the survey.

Category	Number	Question	Selected for the analysis
Demographics and endometriosis	Q1	How old are you?	Yes
	Q2	Profession: (open‐ended question)	No
	Q3	Educational background:	Yes
	Q4A	In which stage you have been diagnosed with endometriosis?	Yes
	Q4B	Since how long you are experiencing symptoms of endometriosis? (open‐ended question)	No
	Q5	How many years you took to get yourself diagnosed with endometriosis?	Yes
	Q6	Which drugs do you take for endometriosis? (open‐ended question)	No
Oral cavity disorders	Q7	Have you experienced any changes regarding your oral cavity after taking these medications?	Yes
	Q8A	Do you have bleeding from gums?	Yes
	Q8B	Is the gum bleeding cyclic?	Yes
	Q8C	Is there any relation you have observed between your bleeding gum and taking endometriosis medications?	Yes
	Q9A	Are your gums swollen and tender?	Yes
	Q9B	Is there any relation you have observed between swollen and tender gums and taking endometriosis medications?	Yes
	Q10	Do you think you are suffering from periodontitis?	Yes
	Q11	How will you describe the health of your teeth and gums?	Yes
	Q12	Have you undergone root planning sessions ever?	Yes
	Q13	Have you noticed the mobility of one or more teeth not caused by trauma?	Yes
	Q14	Has your dentist or hygienist ever told you that you have bone loss around your teeth?	Yes
	Q15A	Do you feel that one or more teeth might require treatment?	Yes
	Q15B	How long has it been since your last professional dental cleaning?	Yes
	Q16	How many times have you used dental floss in the past week? (open‐ended question)	No
	Q17	How many times have you used mouthwash in the past week? (open‐ended question)	No
	Q18	Have you felt dental sensitivity since your diagnosis of endometriosis?	Yes
	Q19	Do you have recurrent canker sores, herpes, or other oral lesions?	Yes
	Q20	Do you think your mouth is dry?	Yes
	Q21	Do you have a burning sensation in your tongue or throughout your mouth?	Yes
	Q22	Since your diagnosis of endometriosis, have you noticed changes on the surface of your tongue?	Yes
	Q23A	From the moment you were diagnosed with endometriosis, have you noticed if your oral disorders have worsened (gingivitis, periodontitis, bleeding gums, canker sores, and dry mouth) or‐if treated‐have not been solved?	Yes
	Q23B	In case of a positive response, report what has been suggested so far for your oral disorders? (open‐ended question)	No
Medical history	Q23C	Does autoimmune pathology affect you‐ for example, Sjögren's syndrome, rheumatological diseases, etc.?	Yes
	Q23D	If the answer to this question is YES, then state the autoimmune pathologies from which you suffer? (open‐ended question)	No
	Q24	Is your dentist and/or dental hygienist aware of your condition?	Yes
Health, wellness and lifestyle	Q25	Do your family/friends tend to downplay your symptoms?	Yes
	Q26A	Has your quality of life been affected by this condition?	Yes
	Q26B	How has this condition impacted your quality of life? (open‐ended question)	No
	Q26C	Before receiving the diagnosis, what was your fruit and vegetable consumption?	Yes
	Q27	Before receiving the diagnosis, what was your alcohol consumption?	Yes
	Q28A	Have you changed your diet after being diagnosed with endometriosis?	Yes
	Q28B	How has your diet changed? (open‐ended question)	No
	Q29A	Pilates, yoga, aerobics, brisk walking, or running around the block, etc.?	Yes
	Q29B	Are you aware that endometriosis can also be indirectly related to pelvic floor muscle disorders?	Yes
	Q30	If you have felt pain during sexual intercourse, describe it:	Yes
	Q31	If you have had surgery for endometriosis and you had pain with intercourse, is this symptom different now than it was before the surgery?	Yes
	Q32	Is it clear to you that physical therapy and rehabilitation are available for symptoms of pelvic floor dysfunction?	Yes
	Q33	Any other considerations or information you feel helpful: (open‐ended question)	No

### 2.1 Statistical Analysis

The descriptive statistical analysis of the survey response was presented using frequencies and percentages. In the comparison between the stages of endometriosis, the chi‐square test or Fisher's exact test was used, as appropriate.

To evaluate the possible relationship between endometriosis and the perception of periodontal diseases, a score was created to summarize on a numerical scale the responses to survey questions regarding the perception of oral cavity disorders. This score was obtained using Multiple Correspondence Analysis (MCA), which was applied to explore relationships between answers. Only the questions with a *p*‐value < 0.25 in the univariate analysis were included in the MCA to ensure the most relevant variables contributed to the score. The answers to the questions were labeled as “yes,” “no,” and “I don't know” (abbreviated as “idk”). In the case of question Q11, a grade was assigned to the responses as follows: “Idk” (I don't know), “Grd0” (Insufficient), “Grd1” (Average), “Grd2” (Good), “Grd3” (Very Good), and “Grd4” (Perfect). Cluster analysis using the k‐means method was performed to identify distinct response patterns. The first MCA dimension was used to calculate a score (Periodontal Diseases Perception Score—PDP score) that increases as the perception of periodontal diseases becomes stronger.

The Kolmogorov–Smirnov test was used to verify the assumption of normality for PDP score and was described using the mean and standard deviation. A multivariate general linear model was used to analyze the relationship between the PDP score and endometriosis stages, considering age and years before receiving the diagnosis of endometriosis as adjustment factors. Both in the comparison of proportions and in the generalized linear model, pairwise multiple comparisons were performed by adjusting the *p*‐value using Tukey's method. A two‐tailed *p*‐value less than 0.05 was considered statistically significant. All analyses were performed using SAS software version 9.4.

## 3 Results

A total of 4072 responses were collected and investigated. 961 women (23.6%) self‐reported stage I of the endometriosis; 771 women (19.0%) reported stage II; 829 women (20.4%) reported stage III; and 1511 women (37.1%) reported stage IV.

### 3.1 Demographics, Endometriosis and Medical History Characteristics

The severity of endometriosis increased significantly with age. Women over 35 years old, who represented only 40.7% at stage I, accounted for 73.0% at stage IV. Educational levels reported by women were significantly different across endometriosis stages. In stages III and IV, the percentage of women with a high school diploma was higher (52.1%) compared to stage I, where women with a university degree prevailed (55.1%). Diagnostic delay was also correlated with the stage of the disease. At stage IV, 47.6% of women reported a diagnostic delay of over 6 years, compared to 28.0% at stage I. Additionally, 18.6% of women at stage IV reported autoimmune diseases, a rate that was significantly higher than the 14.3% at stage I. Only 23.8% of women at stage I reported that their dentist or dental hygienist was aware of their condition. This percentage increased as the disease progressed: 27.8% at stage II, 35.0% at stage III, and 42.0% at stage IV. A high percentage of participants indicated that their symptoms were often minimized or dismissed by family members and close acquaintances, particularly at stages II and III. Meanwhile, the majority of women across all stages felt that the disease influenced their quality of life, with rates increasing significantly from 80.0% at stage I to 93.6% at stage IV (Table 2).

**TABLE 2:** Comparison of responses to questions on demographics, endometriosis, and medical history characteristics by stage of endometriosis.

Questions	Endometriosis Stages					Significant paired comparisons^a^
	Stage I (*n* = 961)	Stage II (*n* = 771)	Stage III (*n* = 829)	Stage IV (*n* = 1511)	*p*	
Educational level						
High School Graduate	432 (45.0%)	387 (50.2%)	432 (52.1%)	787 (52.1%)	**0.0030**	I vs. III; I vs. IV
University Degree	529 (55.1%)	384 (49.8%)	397 (47.9%)	724 (47.9%)		
Age groups (years)						
Aged 15–25	127 (13.2%)	100 (13.0%)	60 (7.2%)	45 (3.0%)	**< 0.0001**	I vs. III; I vs. IV; II vs. III; II vs. IV; II vs. IV
Aged 26–35	443 (46.1%)	308 (40.0%)	292 (35.2%)	362 (24.0%)		
Aged 36–45	321 (33.4%)	284 (36.8%)	381 (46.0%)	833 (55.1%)		
Aged > 45	70 (7.3%)	79 (10.3%)	96 (11.6%)	271 (17.9%)		
Delay in diagnosis (years)						
< 1	327 (34.0%)	215 (27.9%)	180 (21.7%)	292 (19.3%)	**< 0.0001**	I vs. III; I vs. IV; II vs. III; II vs. IV
1–3	229 (23.8%)	194 (25.2%)	176 (21.3%)	277 (18.3%)		
4–6	136 (14.2%)	117 (15.2%)	124 (15.0%)	223 (14.8%)		
> 6	269 (28.0%)	245 (31.8%)	348 (42.0%)	719 (47.6%)		
Q23C						
No	822 (85.7%)	635 (82.6%)	672 (81.3%)	1229 (81.4%)	**0.0287**	I vs. IV
Yes	137 (14.3%)	134 (17.4%)	155 (18.7%)	281 (18.6%)		
Q24						
No	731 (76.2%)	555 (72.2%)	538 (65.1%)	876 (58.0%)	**< 0.0001**	I vs. III; I vs. IV; II vs. III; II vs. IV; III vs. IV
Yes	228 (23.8%)	214 (27.8%)	289 (35.0%)	634 (42.0%)		

*Note:* In bold *p*‐values < 0.05.

^a^ Adjusted by multiple comparison.

### 3.2 Oral Health Disorders

At stage I, 15.3% of women reported noticing changes in their oral health after taking medication, a percentage that increased in later stages (25.2% at stage II to 30.3% at stage IV). There was no correlation between general gum bleeding and the stage of the disease. However, when gum bleeding was linked to the use of endometriosis medications, a higher percentage of women reported this symptom at stage IV (9.2%) compared to stages I (4.1%) and II (6.1%). At stage III, 53.1% of women reported sore gums, compared to 45.4% at stage I. The differences between stage I and later stages became statistically significant when the survey question linked sore gums to medication use for endometriosis. When asked, “Did you think you had periodontitis?” 13.3% of women at stage III responded affirmatively, compared to 8.3% at stage I. Perceptions of dental and gum health were also significantly different. At stage I, 14.4% of women considered their oral health to be “poor,” compared to 22.1% at stage III and 19.2% at stage IV. Women at stage I reported a significantly lower frequency of non‐trauma‐related tooth mobility and bone loss around teeth compared to those at stage IV. At stage III, 55.6% of women believed their teeth needed treatment, compared to 46.5% at stage I. Additionally, 43.8% of women at stages III and IV reported dental sensitivity, compared to 36.9% at stage I. Statistically significant differences emerged between stages I and III regarding higher frequencies of dry mouth (51.5% vs. 42.2%) and burning sensations in the tongue or oral cavity (21.5% vs. 16.4%). Women at stage IV more frequently reported noticing changes in the tongue surface after their endometriosis diagnosis (19.5% vs. 13.4% at stage I). Overall, the frequency of women noticing worsening oral health problems (gingivitis, periodontitis, gum bleeding, mouth ulcers, dry mouth) or unresolved issues post‐treatment increased from 19.75% at stage I to 26.69% at stage IV (Table 3).

**TABLE 3:** Comparison of responses to questions on oral cavity disorders by stage of endometriosis.

Questions	Endometriosis stages					Significant paired comparisons
	Stage I (*n* = 961)	Stage II (*n* = 771)	Stage III (*n* = 829)	Stage IV (*n* = 1511)	*p*	
Q7						
No	810 (84.5%)	577 (74.8%)	600 (72.6%)	1052 (69.7%)	**< 0.0001**	I vs. II; I vs. III; I vs. IV; II vs. IV
Yes	149 (15.5%)	194 (25.2%)	227 (27.5%)	458 (30.3%)		
Q8A						
No	498 (51.9%)	390 (50.6%)	386 (46.7%)	774 (51.3%)	0.1146	
Yes	461 (48.1%)	381 (49.4%)	441 (53.3%)	736 (48.7%)		
Q8B						
No	855 (89.9%)	680 (88.3%)	715 (87.1%)	1343 (89.5%)	0.2160	
Yes	96 (10.1%)	90 (11.7%)	106 (12.9%)	158 (10.5%)		
Q8C						
No	920 (95.9%)	724 (93.9%)	771 (93.3%)	1371 (90.8%)	**< 0.0001**	I vs. IV; II vs. IV
Yes	39 (4.1%)	47 (6.1%)	55 (6.7%)	139 (9.2%)		
Q9A						
No	524 (54.6%)	395 (51.2%)	388 (46.9%)	750 (49.6%)	**0.0098**	I vs. III
Yes	435 (45.4%)	376 (48.8%)	439 (53.1%)	760 (50.3%)		
Q9B						
No	917 (95.6%)	712 (92.4%)	761 (92.1%)	1341 (88.8%)	**< 0.0001**	I vs. II; I vs. III; I vs. IV; II vs. IV
Yes	42 (4.4%)	59 (7.7%)	65 (7.9%)	169 (11.2%)		
Q10						
No	487 (50.8%)	357 (46.3%)	353 (42.7%)	746 (49.4%)	**0.0002**	I vs. III; I vs. IV; III vs. IV
I don't know	392 (40.9%)	326 (42.3%)	364 (44.0%)	570 (37.8%)		
Yes	80 (8.3%)	88 (11.4%)	110 (13.3%)	194 (12.9%)		
Q11						
I don't know	22 (2.3%)	20 (2.6%)	22 (2.7%)	38 (2.5%)	**0.0027**	I vs. III; I vs. IV
Insufficient	138 (14.4%)	140 (18.2%)	183 (22.1%)	290 (19.2%)		
Average	370 (38.6%)	304 (39.4%)	342 (41.4%)	610 (40.4%)		
Good	257 (26.8%)	194 (25.2%)	181 (21.9%)	352 (23.3%)		
Very good	137 (14.3%)	96 (12.5%)	84 (10.2%)	184 (12.2%)		
Perfect	35 (3.7%)	17 (2.2%)	15 (1.8%)	36 (2.4%)		
Q12						
No	830 (86.6%)	639 (82.9%)	686 (83.0%)	1245 (82.5%)	0.1140	I vs. IV
I don't know	77 (8.0%)	81 (10.5%)	84 (10.2%)	174 (11.5%)		
Yes	52 (5.4%)	51 (6.6%)	57 (6.9%)	91 (6.0%)		
Q13						
No	768 (80.1%)	575 (74.6%)	596 (72.1%)	1098 (72.7%)	**0.0001**	I vs. II; I vs. III; I vs. IV
I don't know	47 (4.9%)	56 (7.3%)	63 (7.6%)	84 (5.6%)		
Yes	144 (15.0%)	140 (18.2%)	168 (20.3%)	328 (21.7%)		
Q14						
No	790 (82.4%)	577 (74.8%)	641 (77.5%)	1181 (78.2%)	**< 0.0001**	I vs. II; I vs. IV
I don't know	81 (8.5%)	72 (9.3%)	78 (9.4%)	101 (6.7%)		
Yes	88 (9.2%)	122 (15.8%)	108 (13.1%)	228 (15.1%)		
Q15A						
No	405 (42.2%)	287 (37.2%)	296 (35.8%)	601 (39.8%)	**0.0058**	I vs. III
I don't know	108 (11.3%)	71 (9.2%)	71 (8.6%)	134 (8.9%)		
Yes	446 (46.5%)	413 (53.6%)	460 (55.6%)	775 (51.3%)		
Q15B						
3 months	259 (27.2%)	217 (28.2%)	233 (28.4%)	438 (29.2%)	0.2745	
6 months	190 (20.0%)	156 (20.3%)	132 (16.1%)	286 (19.1%)		
1 year	191 (20.1%)	175 (22.7%)	193 (23.5%)	316 (21.1%)		
More than 1 year	311 (32.7%)	222 (28.8%)	263 (32.0%)	461 (30.7%)		
Q18						
No	605 (63.1%)	444 (57.6%)	465 (56.2%)	849 (56.2%)	**0.0041**	I vs. III; I vs. IV
Yes	354 (36.9%)	327 (42.4%)	362 (43.8%)	661 (43.8%)		
Q19						
No	538 (56.1%)	417 (54.1%)	422 (51.0%)	828 (54.8%)	0.1721	
Yes	421 (43.9%)	354 (45.9%)	405 (49.0%)	682 (45.2%)		
Q20						
No	526 (54.9%)	393 (51.0%)	401 (48.5%)	800 (53.0%)	**0.0443**	I vs. III
Yes	433 (45.2%)	378 (49.0%)	426 (51.5%)	710 (47.0%)		
Q21						
No	802 (83.6%)	624 (80.9%)	649 (78.5%)	1204 (79.7%)	**0.0321**	I vs. III
Yes	157 (16.4%)	147 (19.1%)	178 (21.5%)	306 (20.3%)		
Q22						
No	831 (86.7%)	636 (82.5%)	687 (83.1%)	1215 (80.5%)	**0.0012**	I vs. IV
Yes	128 (13.4%)	135 (17.5%)	140 (16.9%)	295 (19.5%)		
Q23A						
No	770 (80.3%)	573 (74.3%)	606 (73.3%)	1104 (73.1%)	**0.0003**	I vs. II; I vs. III; I vs. IV
Yes	189 (19.7%)	198 (25.7%)	221 (26.7%)	406 (26.9%)		

*Note:* In bold *p*‐values < 0.05.

^a^ Adjusted by multiple comparison.

### 3.3 Health, Wellness, and Lifestyle Factors

Women at stages II, III, and IV more frequently reported changing their diet after being diagnosed with endometriosis. They were also more frequently aware of the indirect link between the condition and pelvic floor muscle disorders. Finally, women at stages II, III, and IV reported higher rates of deep vaginal pain and changes in vaginal pain during intercourse following surgical intervention. Awareness of the positive effects of physiotherapy and rehabilitation for pelvic floor dysfunction symptoms also increased significantly, from 59.0% at stage I to 74.9% at stage IV (Table 4).

**TABLE 4:** Comparison of responses to questions on health, wellness, and lifestyle characteristics by stage of endometriosis.

Health, wellness and lifestyle	Endometriosis stages				*p*	Significant paired comparisons
	Stage I (*n* = 961)	Stage II (*n* = 771)	Stage III (*n* = 829)	Stage IV (*n* = 1511)		
Q25						
No	536 (55.9%)	383 (49.8%)	416 (50.3%)	864 (57.2%)	**0.0005**	I vs. II; II vs. IV; III vs. IV
Yes	423 (44.1%)	386 (50.2%)	411 (49.7%)	646 (42.8%)		
Q26A						
No	192 (20.0%)	86 (11.2%)	57 (6.9%)	96 (6.4%)	**< 0.0001**	I vs. II; I vs. III; I vs. IV; II vs. III; II vs. IV
Yes	767 (80.0%)	683 (88.8%)	770 (93.1%)	1414 (93.6%)		
Q26C						
I don't consume it, rarely	168 (17.6%)	131 (17.1%)	136 (16.5%)	276 (18.3%)	0.3066	
1–2 portions a day	594 (62.1%)	444 (58.0%)	517 (62.6%)	905 (60.1%)		
Between 3 and 4 portions a day	159 (16.6%)	166 (21.7%)	144 (17.4%)	266 (17.7%)		
At least 5 portions or more a day	36 (3.8%)	25 (3.3%)	29 (3.5%)	59 (3.9%)		
Q27						
Never, rarely	517 (54.0%)	420 (54.8%)	508 (61.5%)	919 (61.0%)	**0.0003**	I vs. III; I vs. IV
1–3 units per week	380 (39.7%)	282 (36.8%)	275 (33.3%)	500 (33.2%)		
4–6 units per week	54 (5.6%)	46 (6.0%)	33 (4.0%)	69 (4.6%)		
Every Day 1 or more per day	6 (0.6%)	18 (2.3%)	10 (1.2%)	18 (1.2%)		
Q28A						
No	424 (44.3%)	264 (34.5%)	258 (31.2%)	484 (32.1%)	**< 0.0001**	I vs. II; I vs. III; I vs. IV
Yes	533 (55.7%)	502 (65.5%)	568 (68.8%)	1022 (67.9%)		
Q29A						
I have not practiced any type of physical activity or sports	213 (22.3%)	156 (20.4%)	184 (22.3%)	320 (21.3%)	0.2489	
Less frequently	239 (25.0%)	165 (21.5%)	179 (21.7%)	353 (23.5%)		
1–2 times a week	289 (30.2%)	231 (30.2%)	239 (28.9%)	449 (29.9%)		
3–4 times a week	161 (16.8%)	159 (20.8%)	183 (22.2%)	294 (19.6%)		
5 or more times a week	54 (5.7%)	55 (7.2%)	41 (5.0%)	87 (5.8%)		
Q29B						
No	467 (48.9%)	321 (41.9%)	299 (36.2%)	471 (31.3%)	**< 0.0001**	I vs. II; I vs. III; I vs. IV; II vs. IV
Yes	489 (51.2%)	445 (58.1%)	527 (63.8%)	1032 (68.7%)		
Q30						
At the vaginal entrance	157 (16.4%)	114 (14.9%)	101 (12.2%)	190 (12.6%)	**< 0.0001**	I vs. II; I vs. III; I vs. IV
In both cases	322 (33.7%)	290 (37.9%)	335 (40.6%)	637 (42.4%)		
I don't have sexual relations	91 (9.5%)	39 (5.1%)	42 (5.1%)	55 (3.7%)		
Deeply	386 (40.4%)	323 (42.2%)	348 (42.1%)	621 (41.3%)		
Q31						
No	222 (23.2%)	253 (33.0%)	324 (39.2%)	661 (44.0%)	**< 0.0001**	I vs. II; I vs. III; I vs. IV; II vs. III; II vs. IV; III vs. IV
I have not undergone any surgeries	606 (63.4%)	356 (46.5%)	237 (28.7%)	159 (10.6%)		
Yes	128 (13.4%)	157 (20.5%)	265 (32.1%)	683 (45.4%)		
Q32						
No	392 (41.0%)	291 (38.0%)	257 (31.1%)	377 (25.1%)	**< 0.0001**	I vs. III; I vs. IV; II vs. III; II vs. IV; III vs. IV
Yes	564 (59.0%)	475 (62.0%)	569 (68.9%)	1126 (74.9%)		

*Note:* In bold *p*‐values < 0.05.

^a^ Adjusted by multiple comparison.

### 3.4 Periodontal Diseases Perception Score – PDP Score

**FIGURE 1:**
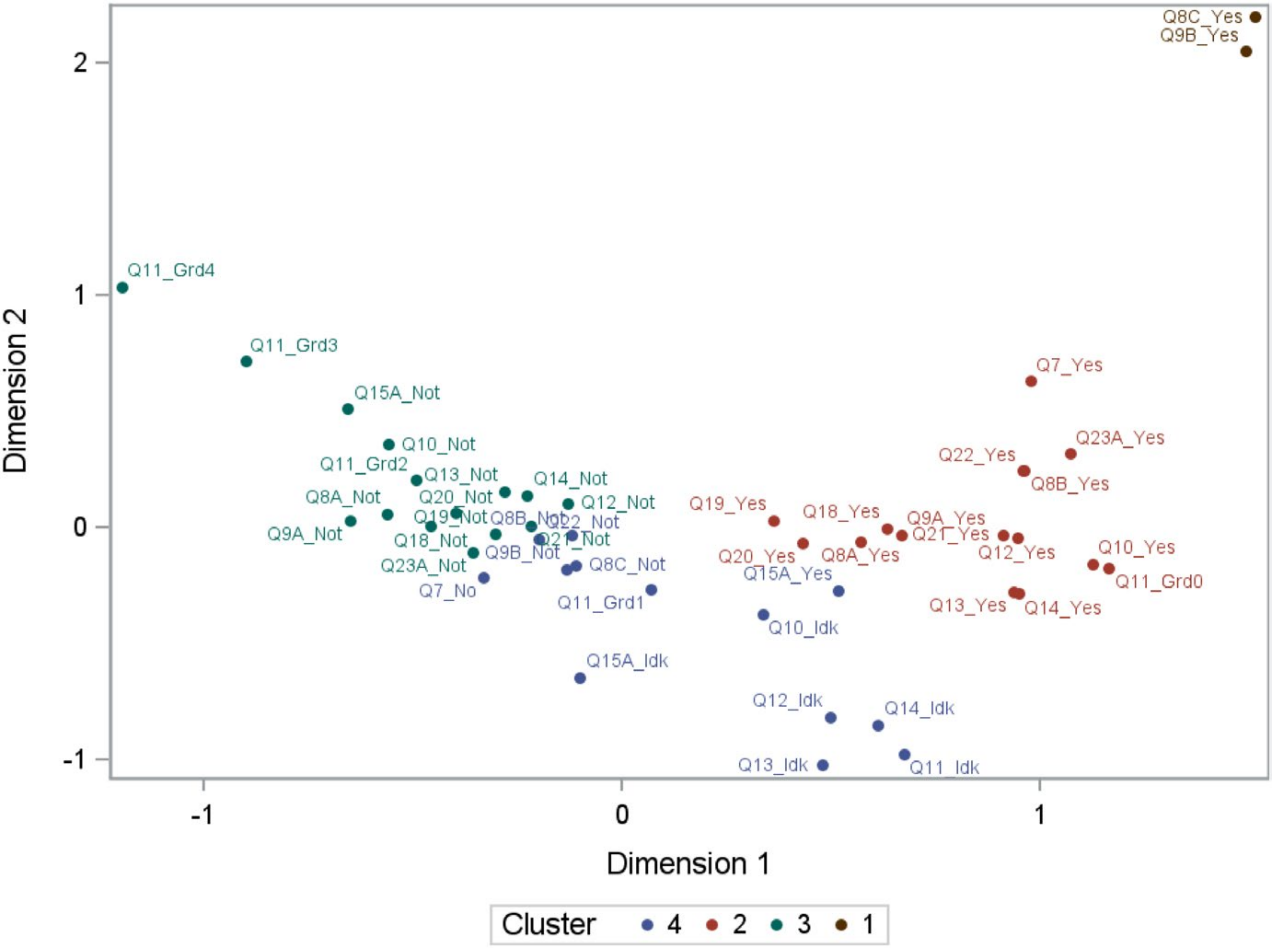
Clusters of responses based on the two dimensions of multiple correspondence analysis. Notably, Dimension 1 discriminated between two major response categories: Those indicative of a negative perception of oral health (clusters 1 and 2) and those suggesting a non‐negative perception (cluster 3). For this reason, weights corresponding to the values of the first dimension were assigned to the responses of everyone. Since cluster 4 primarily included responses where patients answered, “I don't know,” these responses were assigned a weight of 0 in the score calculation (Figure S1). Based on these findings, the PDP score was computed for each patient by summing the assigned weights.

Multiple Correspondence Analysis (MCA) applied to explore relationships between answers to the questionnaire revealed that the first dimension explains 86% of the total variance, while the first and second dimensions together account for 89% of the total variability. These two dimensions were used as coordinates to construct a plot (Figure 1), where proximity indicates similarity between categories. The k‐means cluster analysis identified four distinct clusters, represented by different colors in the figure.

Multivariate general linear model was performed to examine the relationship between the PDP score and the endometriosis stage, adjusted by age class and delays in receiving the diagnosis. Endometriosis stage was a statistically significant predictor, with Stage II, III, and IV showing the largest coefficient (*β* = 0.76, *p* < 0.0001; *β* = 0.96, *p* < 0.0001; *β* = 0.66, *p* < 0.0001), indicating that patients in these stages reported higher PDP scores compared to those in Stage I. The class of age had a statistically significant effect on the PDP score: women over 45 years of age showed the largest coefficient compared to women aged between 15 and 25 years (*β* = 0.76, *p* < 0.0001) meaning that older women tended to report a higher score of perception of periodontal diseases, which is consistent with the well‐established evidence that both the prevalence and severity of periodontal disease increase with age. Prominent forms of periodontal disease are, in fact, very rare in individuals under the age of 25. Years passed before receiving the endometriosis diagnosis was found to be positively associated with the PDP score, suggesting that longer delays in receiving the diagnosis were associated with higher scores of the perception of periodontal diseases (Table 5).

**TABLE 5:** Results of the multivariate general linear model performed to examine the relationship between the PDP score and the endometriosis stage, adjusted by age class and delays in receiving the diagnosis.

Parameter	Coefficient (SE)	*p*
Stages of endometriosis		
I stage	ref.	
II stage	0.76 (0.21)	**< 0.0001**
III stage	0.96 (0.20)	**< 0.0001**
IV stage	0.66 (0.18)	**< 0.0001**
Age class (years)		
Aged 15–25	ref.	
Aged 26–35	−0.23 (0.26)	0.376
Aged 36–45	0.23 (0.26)	0.372
Aged > 45	0.76 (0.31)	**0.014**
Delay in diagnosis (years)		
< 1	ref.	
1–3	1.07 (0.2)	**< 0.0001**
4–6	1.33 (0.22)	**< 0.0001**
> 6	1.71 (0.17)	**< 0.0001**

*Note:* In bold *p*‐values < 0.05.

Abbreviation: SE, standard error.

**FIGURE 2:**
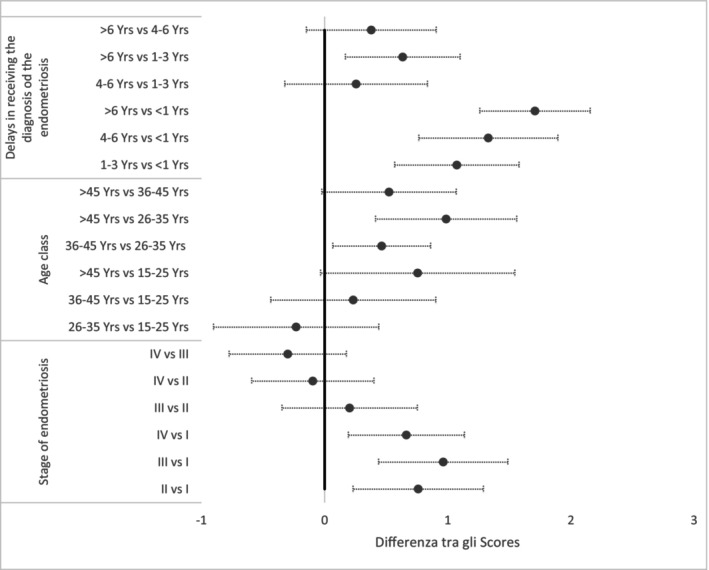
Forest plot of the differences of the PDP scores estimates from the multivariate general linear model.

Patients diagnosed after more than 6 years had a significantly higher PDP score compared to those diagnosed within 1 year (Δ = 1.71, 95% CI: 1.26–2.16) (Figure 2). Similarly, a diagnostic delay of 4–6 years was associated with a higher PDP score compared to < 1 year (Δ = 1.33, 95% CI: 0.76–1.89). Regarding disease severity, patients with stage III and IV endometriosis showed a higher PDP score compared to stage I (Δ = 0.96, 95% CI: 0.44–1.49 for stage III; Δ = 0.66, 95% CI: 0.19–1.14 for stage IV). Differences among age groups were less pronounced, with no significant variations in PDP scores between most comparisons.

## 4 Discussion

The analysis of the gathered data provides a thorough understanding of the relationship between endometriosis and the perception of oral health, revealing how different stages of the disease affect both the quality of life and the dental health of individuals (Yalçın Bahat et al. 2022; Capezzuoli et al. 2022).

Findings emphasize the significant relationship between the advancement of endometriosis and increased negative perceptions about oral health, which were measured by the Periodontal Diseases Perception (PDP) score. Notably, the analysis revealed that women diagnosed with advanced stages of endometriosis (II, III, and IV) exhibited a heightened perception of periodontal diseases, suggesting potential oral health implications of this chronic condition (Sobstyl et al. 2023; Grandi et al. 2019).

A particularly significant finding is the progressive increase in the PDP score in relation to the endometriosis severity, further emphasizing the systemic impact of the disease on oral health. Women classified in stages II, III, and IV exhibited significantly higher periodontal disease perception (PDP) scores than those in stage I, with no substantial difference observed among the advanced stages. This suggests a strong relation between endometriosis progression and the perceived deterioration of periodontal health. Although the underlying mechanisms are still incompletely defined, they likely involve systemic effects of the disease and its treatments, encompassing chronic inflammation, hormonal dysregulation, and potential pharmacological impacts on oral tissues. As endometriosis advances, exacerbation of oral health‐related symptoms, such as gingival bleeding, xerostomia, and dentin hypersensitivity, may arise due to immune alterations, endocrine disturbances, and prolonged medication exposure (Shigesi et al. 2019; Zervou et al. 2024; Chao et al. 2022).

Moreover, multivariate general linear modeling found age and diagnostic delay as significant contributors to PDP scores. Older women and those experiencing prolonged diagnostic latency reported a more pronounced decline in perceived oral health, underscoring the multifaceted burden of advanced endometriosis (van Stein et al. 2023).

These findings highlight the interplay between disease chronicity and heightened awareness of oral health complications, further emphasizing the need for an integrated, multidisciplinary approach in the management of patients with advanced endometriosis (McGrath et al. 2023).

The observed relation between delayed diagnosis and deteriorating oral health perception is particularly concerning, as prolonged diagnostic latency may extend exposure to both disease‐related and treatment‐induced factors, including stress, that negatively impact periodontal integrity (Macrì et al. 2024).

The study revealed a progressive escalation of oral complications—manifesting as gingival pain, bleeding, and alterations in tongue morphology—parallel to disease advancement. Notably, pharmacological management of endometriosis, particularly in stage IV, was strongly associated with increased gingival bleeding, a finding corroborated by existing literature, which attributes this phenomenon to the effects of hormonal therapies on periodontal tissues. Additionally, other oral manifestations, including dental hypersensitivity, xerostomia, and a burning sensation of the tongue, exhibited greater prevalence in the later stages of the disease (Hartner et al. 2023; Tourny et al. 2023).

These findings suggest an intricate interplay between systemic inflammatory responses, endocrine dysregulation, and long‐term pharmacological exposure, all of which could contribute to the exacerbation of oral health disturbances in patients with advanced endometriosis (Gajbhiye 2023).

The findings of this study highlight a growing awareness among women regarding the potential link between endometriosis and poor oral health (Teal and Edelman 2021).

However, this awareness is not yet mirrored among dental professionals, whose knowledge of the condition remains relatively limited. In the early stages of the disease, only 23.8% of women reported that their dentist or dental hygienist was aware of their diagnosis, with this percentage increasing as the disease progressed. This disparity highlights the urgent need for enhanced interdisciplinary communication between patients, dental practitioners, and dental hygienists, particularly for those in advanced stages of endometriosis. Dental professionals should adopt a more proactive role in recognizing and managing the complex oral health challenges that these patients face, as their condition necessitates specialized and tailored care (Fiorillo 2019; Gaffar et al. 2022; Schmalz et al. 2023).

Beyond oral health, women with more severe endometriosis exhibited significant lifestyle adaptations, including dietary modifications and heightened awareness of pelvic floor dysfunction, suggesting a more comprehensive perception of the disease's systemic impact (Gutke et al. 2021).

A similar pattern of heightened awareness may extend to oral health concerns. However, the elevated prevalence of dental hypersensitivity and periodontal diseases in advanced stages indicates a pressing need for more vigilant and preventive dental care in this population (Clavagnier 2015).

These findings emphasize the need for early diagnosis and multidisciplinary management of endometriosis, advocating for integrative approaches that incorporate both gynecological and dental expertise. Periodontal health maintenance should be regarded as a crucial component of patient management, particularly in advanced disease stages, where oral manifestations become more pronounced (Wu et al. 2024; Crump et al. 2024).

Furthermore, the increased recognition and documentation of oral health complications among women with endometriosis should prompt further investigation into the intricate relation between endometriosis and periodontal disease (Jin et al. 2024; Thomas et al. 2018; Kavoussi et al. 2009).

This calls for the development of evidence‐based guidelines specifically addressing the oral healthcare needs of women with this chronic condition (Machado et al. 2020).

Ultimately, this study underscores the necessity for continued research into the underlying pathophysiological mechanisms linking endometriosis and periodontal disease. Raising awareness among healthcare providers and patients about the potential oral health ramifications of endometriosis is imperative to ensure that affected women receive comprehensive care, encompassing both their reproductive and oral health needs. Future research should prioritize elucidating these mechanistic pathways and evaluating targeted interventions aimed at mitigating the oral health consequences associated with endometriosis (Halawani et al. 2024).

### 4.1 Demographics and Medical History

The results support an association between endometriosis severity and patient age, with a higher prevalence of women over 35 in advanced disease stages (Comptour et al. 2024).

This observation suggests that endometriosis progression may be driven by cumulative factors over time, including persistent hormonal dysregulation, chronic inflammation, and delayed diagnosis. Furthermore, the elevated incidence of comorbidities such as irritable bowel syndrome and autoimmune disorders in these patients highlights the systemic nature of endometriosis, reinforcing its multifaceted pathophysiology and the necessity for an integrative therapeutic approach.

### 4.2 Impact on Quality of Life

A particularly striking aspect of this study is the profound, multidimensional impact of endometriosis on patients' quality of life (Nnoaham et al. 2011).

Across all disease stages, participants reported substantial pain and fatigue, with symptom severity escalating in advanced cases. The intricate interplay between chronic pain and psychological distress was evident, as many individuals exhibited symptoms of anxiety and depression, further exacerbating the burden of daily functioning. These findings reinforce the biopsychosocial complexity of endometriosis, aligning with existing literature that underscores its far‐reaching systemic implications.

### 4.3 Oral Health Perception and Symptoms

These findings highlight a largely overlooked aspect of endometriosis: its impact on oral health. Patients across all stages reported an increased prevalence of dental hypersensitivity, gingival bleeding, and orofacial pain compared to the general population. Notably, those in advanced stages exhibited a higher incidence of temporomandibular joint (TMJ) dysfunction, suggesting a potential association between chronic systemic inflammation and musculoskeletal involvement. This aligns with emerging research indicating that the inflammatory burden of endometriosis may extend beyond the reproductive system, contributing to oral and temporomandibular disorders. These insights underscore the importance of an interdisciplinary approach to patient care, integrating dentistry into the broader management of endometriosis (Kaushik et al. 2020).

### 4.4 Potential Mechanisms

Multiple pathophysiological mechanisms may underlie the association between endometriosis and oral health disturbances. Chronic systemic inflammation, a defining feature of endometriosis, likely exacerbates periodontal disease and other oral pathologies by promoting a pro‐inflammatory microenvironment. Additionally, hormonal dysregulation—particularly estrogenic imbalance—may influence oral tissue homeostasis, given the presence of estrogen receptors in gingival and mucosal tissues, which modulate their response to injury and infection (Marquardt et al. 2023; Boyapati et al. 2021).

Furthermore, the psychological burden of a chronic illness, including heightened stress levels, may contribute to oral health deterioration, as stress is a well‐documented risk factor for conditions such as bruxism, periodontal inflammation, and immune dysregulation within the oral cavity.

### 4.5 Implications for Clinical Practice

This study highlights the imperative for a multidisciplinary approach in the management of endometriosis, emphasizing the integration of gynecologists, dental specialists, and mental health professionals to comprehensively address the heterogeneous symptomatology of affected individuals (Mazza et al. 2023). Routine oral health assessments and individualized therapeutic strategies may play a pivotal role in mitigating the oral manifestations frequently observed in this patient population, ultimately enhancing their overall quality of life. Furthermore, heightened awareness among healthcare providers regarding the systemic and multifactorial nature of endometriosis could facilitate earlier detection and the implementation of a more holistic and patient‐centered treatment paradigm.

### 4.6 Limitations of the Study

This study presents limitations that should be acknowledged when interpreting the findings. First, the cross‐sectional design, based on self‐reported data from an online questionnaire, does not allow for causal inferences or the establishment of temporal relationships between endometriosis and periodontal disease. The use of self‐reported periodontal conditions, while practical for large‐scale data collection, may be subject to recall bias and does not replace objective clinical assessments.

Moreover, participants were recruited through Facebook support groups for women with endometriosis, which, although useful for reaching a large and engaged population, may introduce selection bias, as it may not fully represent the broader demographic and clinical variability of all women affected by the condition. In particular, individuals with more severe symptoms or greater disease awareness may have been more inclined to participate.

Finally, although the questionnaire was developed in collaboration with a multidisciplinary team and validated by experts, it was not previously standardized or psychometrically validated in similar studies, which may affect the reproducibility of the results.

## 5 Conclusion

This study highlights the intricate interplay between endometriosis, oral health, and overall quality of life, emphasizing the need for a multidisciplinary and integrative approach to patient care. Recognizing these interconnections allows healthcare professionals to implement more comprehensive strategies, ultimately improving clinical outcomes and patient well‐being. Future research should focus on elucidating the underlying pathophysiological mechanisms linking endometriosis to oral health and developing targeted therapeutic interventions that address both systemic and localized manifestations of the disease.

## Author Contributions

**Maria Teresa Agneta:** conceptualization, investigation, writing – original draft, methodology, validation, visualization, writing – review and editing, supervision, project administration. **Giuseppe D'Albis:** conceptualization, investigation, writing – original draft, methodology, visualization, writing – review and editing, formal analysis, data curation, resources, validation. **Letizia Lorusso:** formal analysis, data curation, resources, software, writing – review and editing. **Nicola Bartolomeo:** software, formal analysis, data curation, writing – review and editing, supervision. **Antonia Abbinante:** investigation, methodology, writing – original draft. **Anna Antonacci:** investigation. **Pietro Signorile:** investigation, conceptualization, methodology, validation, visualization. **Francesco D'Aiuto:** conceptualization, investigation, methodology, validation, writing – review and editing, visualization. **Elisa Mazza:** conceptualization, investigation, writing – original draft, methodology, validation, visualization, writing – review and editing. **Massimo Corsalini:** supervision, project administration, resources. **Saverio Capodiferro:** conceptualization, investigation, writing – original draft, methodology, validation, visualization, writing – review and editing, formal analysis, project administration, supervision.

## Conflicts of Interest

The authors declare no conflicts of interest.

## Supporting information


**Figure S1.** Weights assigned to responses based on the first dimension of multiple correspondence analysis.

## Data Availability

The data that support the findings of this study are available on request from the corresponding author. The data are not publicly available due to privacy or ethical restrictions.

## References

[odi70044-bib-0001] Amro, B. , M. E. Ramirez Aristondo , S. Alsuwaidi , et al. 2022. “New Understanding of Diagnosis, Treatment and Prevention of Endometriosis.” International Journal of Environmental Research and Public Health 19, no. 11: 6725. 10.3390/ijerph19116725. 35682310 PMC9180566

[odi70044-bib-0002] Boyapati, R. , S. A. Cherukuri , R. Bodduru , and A. Kiranmaye . 2021. “Influence of Female Sex Hormones in Different Stages of Women on Periodontium.” Journal of Mid‐Life Health 12, no. 4: 263–266. 10.4103/jmh.jmh_142_21. 35264831 PMC8849144

[odi70044-bib-0003] Capezzuoli, T. , M. Rossi , F. La Torre , S. Vannuccini , and F. Petraglia . 2022. “Hormonal Drugs for the Treatment of Endometriosis.” Current Opinion in Pharmacology 67: 102311. 10.1016/j.coph.2022.102311. 36279764

[odi70044-bib-0004] Chao, Y. H. , C. H. Liu , Y. A. Pan , F. S. Yen , J. Y. Chiou , and J. C. Wei . 2022. “Association Between Endometriosis and Subsequent Risk of Sjögren's Syndrome: A Nationwide Population‐Based Cohort Study.” Frontiers in Immunology 13: 845944. 10.3389/fimmu.2022.845944. 35592328 PMC9110644

[odi70044-bib-0005] Clavagnier, I. 2015. “Preventive Dentistry and Oral Hygiene.” Revue de l'Infirmière 54, no. 211: 49–50. 10.1016/j.revinf.2015.02.019. 26145701

[odi70044-bib-0006] Comptour, A. , C. Figuier , B. Pereira , P. Chauvet , N. Bourdel , and M. Canis . 2024. “Endometriosis: Age at Diagnosis and the Severity of the Disease.” Journal of Gynecology Obstetrics and Human Reproduction 53, no. 5: 102759. 10.1016/j.jogoh.2024.102759. 38467186

[odi70044-bib-0007] Crump, J. , A. Suker , and L. White . 2024. “Endometriosis: A Review of Recent Evidence and Guidelines.” Australian Journal of General Practice 53, no. 1‐2: 11–18. 10.31128/AJGP/04-23-6805. 38316472

[odi70044-bib-0008] Culley, L. , C. Law , N. Hudson , et al. 2013. “The Social and Psychological Impact of Endometriosis on Women's Lives: A Critical Narrative Review.” Human Reproduction Update 19, no. 6: 625–639. 10.1093/humupd/dmt027. 23884896

[odi70044-bib-0009] Evans, S. , S. Fernandez , L. Olive , L. A. Payne , and A. Mikocka‐Walus . 2019. “Psychological and Mind‐Body Interventions for Endometriosis: A Systematic Review.” Journal of Psychosomatic Research 124: 109756. 10.1016/j.jpsychores.2019.109756. 31443810

[odi70044-bib-0010] Fiorillo, L. 2019. “Oral Health: The First Step to Well‐Being.” Medicina (Kaunas, Lithuania) 55, no. 10: 676. 10.3390/medicina55100676. 31591341 PMC6843908

[odi70044-bib-0011] Gaffar, B. , F. A. Farooqi , M. A. Nazir , et al. 2022. “Oral Health‐Related Interdisciplinary Practices Among Healthcare Professionals in Saudi Arabia: Does Integrated Care Exist?” BMC Oral Health 22, no. 1: 75. 10.1186/s12903-022-02113-5. 35300658 PMC8928017

[odi70044-bib-0012] Gajbhiye, R. K. 2023. “Endometriosis and Inflammatory Immune Responses: Indian Experience.” American Journal of Reproductive Immunology 89, no. 2: e13590. 10.1111/aji.13590. 35751585 PMC7615030

[odi70044-bib-0013] Garcia Garcia, J. M. , V. Vannuzzi , C. Donati , C. Bernacchioni , P. Bruni , and F. Petraglia . 2023. “Endometriosis: Cellular and Molecular Mechanisms Leading to Fibrosis.” Reproductive Sciences 30, no. 5: 1453–1461. 10.1007/s43032-022-01083-x. 36289173 PMC10160154

[odi70044-bib-0014] Grandi, G. , F. Barra , S. Ferrero , et al. 2019. “Hormonal Contraception in Women With Endometriosis: A Systematic Review.” European Journal of Contraception & Reproductive Health Care 24, no. 1: 61–70. 10.1080/13625187.2018.1550576. 30664383

[odi70044-bib-0015] Gutke, A. , K. Sundfeldt , and L. De Baets . 2021. “Lifestyle and Chronic Pain in the Pelvis: State of the Art and Future Directions.” Journal of Clinical Medicine 10, no. 22: 5397. 10.3390/jcm10225397. 34830680 PMC8622577

[odi70044-bib-0016] Halawani, S. M. , S. N. Alokaili , A. M. Ajeebi , and P. Koppolu . 2024. “Endometriosis Associated With Periodontal Disease: Two Case Reports.” Journal of Pharmacy & Bioallied Sciences 16, no. Suppl 4: S4169–S4172. 10.4103/jpbs.jpbs_1298_24. 39926878 PMC11805232

[odi70044-bib-0017] Hartner, G. , H. Husslein , L. Kuessel , et al. 2023. “The Latest Advances in the Pharmacological Management of Endometriosis.” Expert Opinion on Pharmacotherapy 24, no. 1: 121–133. 10.1080/14656566.2022.2045274. 35232316

[odi70044-bib-0019] Jin, B. , P. Wang , P. Liu , et al. 2024. “Association Between Periodontitis and Endometriosis: A Bidirectional Mendelian Randomization Study.” Frontiers in Endocrinology 15: 1271351. 10.3389/fendo.2024.1271351. 38487346 PMC10937447

[odi70044-bib-0020] Kaushik, T. , R. Mishra , R. K. Singh , and S. Bajpai . 2020. “Role of Connexins in Female Reproductive System and Endometriosis.” Journal of Gynecology Obstetrics and Human Reproduction 49, no. 6: 101705. 10.1016/j.jogoh.2020.101705. 32018041

[odi70044-bib-0021] Kavoussi, S. K. , B. T. West , G. W. Taylor , and D. I. Lebovic . 2009. “Periodontal Disease and Endometriosis: Analysis of the National Health and Nutrition Examination Survey.” Fertility and Sterility 91, no. 2: 335–342. 10.1016/j.fertnstert.2007.12.075. 18394619 PMC2674278

[odi70044-bib-0022] Koninckx, P. R. , A. Ussia , L. Adamyan , A. Wattiez , V. Gomel , and D. C. Martin . 2019. “Pathogenesis of Endometriosis: The Genetic/Epigenetic Theory.” Fertility and Sterility 111, no. 2: 327–340. 10.1016/j.fertnstert.2018.10.013. 30527836

[odi70044-bib-0023] Machado, V. , J. Lopes , M. Patrão , J. Botelho , L. Proença , and J. J. Mendes . 2020. “Validity of the Association Between Periodontitis and Female Infertility Conditions: A Concise Review.” Reproduction 160, no. 3: R41–R54. 10.1530/REP-20-0176. 32716008

[odi70044-bib-0024] Macrì, M. , G. D'Albis , V. D'Albis , et al. 2024. “Periodontal Health and Its Relationship With Psychological Stress: A Cross‐Sectional Study.” Journal of Clinical Medicine 13, no. 10: 2942. 10.3390/jcm13102942. 38792482 PMC11122378

[odi70044-bib-0025] Marquardt, R. M. , D. N. Tran , B. A. Lessey , M. S. Rahman , and J. W. Jeong . 2023. “Epigenetic Dysregulation in Endometriosis: Implications for Pathophysiology and Therapeutics.” Endocrine Reviews 44, no. 6: 1074–1095. 10.1210/endrev/bnad020. 37409951 PMC10638603

[odi70044-bib-0026] Mazza, E. , E. Troiano , S. Mazza , et al. 2023. “The Impact of Endometriosis on Dietary Choices and Activities of Everyday Life: A Cross‐Sectional Study.” Frontiers in Nutrition 10, no. 22: 1273976. 10.3389/fnut.2023.1273976. 37810932 PMC10559972

[odi70044-bib-0027] McGrath, I. M. , G. W. Montgomery , and S. Mortlock . 2023. “Insights From Mendelian Randomization and Genetic Correlation Analyses Into the Relationship Between Endometriosis and Its Comorbidities.” Human Reproduction Update 29, no. 5: 655–674. 10.1093/humupd/dmad009. 37159502 PMC10477944

[odi70044-bib-0028] Nnoaham, K. E. , L. Hummelshoj , P. Webster , et al. 2011. “Impact of Endometriosis on Quality of Life and Work Productivity: A Multicenter Study Across Ten Countries.” Fertility and Sterility 96, no. 2: 366–373. 10.1016/j.fertnstert.2011.05.090. 21718982 PMC3679489

[odi70044-bib-0029] Schmalz, G. , C. Lenzen , F. Reuschel , et al. 2023. “Lack of Oral Health Awareness and Interdisciplinary Dental Care: A Survey in Patients Prior to Endoprosthesis and Orthopaedic Centres in Germany.” BMC Oral Health 23, no. 1: 92. 10.1186/s12903-023-02793-7. 36782181 PMC9926854

[odi70044-bib-0030] Shigesi, N. , M. Kvaskoff , S. Kirtley , et al. 2019. “The Association Between Endometriosis and Autoimmune Diseases: A Systematic Review and Meta‐Analysis.” Human Reproduction Update 25, no. 4: 486–503. 10.1093/humupd/dmz014. 31260048 PMC6601386

[odi70044-bib-0031] Sobstyl, A. , A. Chałupnik , P. Mertowska , and E. Grywalska . 2023. “How Do Microorganisms Influence the Development of Endometriosis? Participation of Genital, Intestinal and Oral Microbiota in Metabolic Regulation and Immunopathogenesis of Endometriosis.” International Journal of Molecular Sciences 24, no. 13: 10920. 10.3390/ijms241310920. 37446108 PMC10341671

[odi70044-bib-0032] Somigliana, E. , L. Li Piani , A. Paffoni , et al. 2023. “Endometriosis and IVF Treatment Outcomes: Unpacking the Process.” Reproductive Biology and Endocrinology 21, no. 1: 107. 10.1186/s12958-023-01157-8. 37936154 PMC10629090

[odi70044-bib-0034] Teal, S. , and A. Edelman . 2021. “Contraception Selection, Effectiveness, and Adverse Effects: A Review.” Journal of the American Medical Association 326, no. 24: 2507–2518. 10.1001/jama.2021.21392. 34962522

[odi70044-bib-0035] Thomas, V. , A. S. Uppoor , S. Pralhad , D. G. Naik , and P. Kushtagi . 2018. “Towards a Common Etiopathogenesis: Periodontal Disease and Endometriosis.” Journal of Human Reproductive Sciences 11, no. 3: 269–273. 10.4103/jhrs.JHRS_8_18. 30568357 PMC6262667

[odi70044-bib-0036] Tourny, C. , A. Zouita , S. El Kababi , et al. 2023. “Endometriosis and Physical Activity: A Narrative Review.” International Journal of Gynecology & Obstetrics 163, no. 3: 747–756. 10.1002/ijgo.14898. 37345574

[odi70044-bib-0037] Van Niekerk, L. , B. Weaver‐Pirie , and M. Matthewson . 2019. “Psychological Interventions for Endometriosis‐Related Symptoms: A Systematic Review With Narrative Data Synthesis.” Archives of Women's Mental Health 22, no. 6: 723–735. 10.1007/s00737-019-00972-6. 31081520

[odi70044-bib-0038] van Stein, K. , K. Schubert , B. Ditzen , and C. Weise . 2023. “Understanding Psychological Symptoms of Endometriosis From a Research Domain Criteria Perspective.” Journal of Clinical Medicine 12, no. 12: 4056. 10.3390/jcm12124056. 37373749 PMC10299570

[odi70044-bib-0039] Vannuccini, S. , S. Clemenza , M. Rossi , and F. Petraglia . 2022. “Hormonal Treatments for Endometriosis: The Endocrine Background.” Reviews in Endocrine & Metabolic Disorders 23, no. 3: 333–355. 10.1007/s11154-021-09666-w. 34405378 PMC9156507

[odi70044-bib-0040] Wu, J. , J. Li , M. Yan , and Z. Xiang . 2024. “Gut and Oral Microbiota in Gynecological Cancers: Interaction, Mechanism, and Therapeutic Value.” NPJ Biofilms and Microbiomes 10, no. 1: 104. 10.1038/s41522-024-00577-7. 39389989 PMC11467339

[odi70044-bib-0041] Yalçın Bahat, P. , I. Ayhan , E. Üreyen Özdemir , Ü. İnceboz , and E. Oral . 2022. “Dietary Supplements for Treatment of Endometriosis: A Review.” Acta Biomedica Atenei Parmensis 93, no. 1: e2022159. 10.23750/abm.v93i1.11237. PMC8972862 35315418

[odi70044-bib-0042] Zervou, M. I. , B. C. Tarlatzis , G. F. Grimbizis , D. A. Spandidos , T. B. Niewold , and G. N. Goulielmos . 2024. “Association of Endometriosis With Sjögren's Syndrome: Genetic Insights (Review).” International Journal of Molecular Medicine 53, no. 2: 20. 10.3892/ijmm.2024.5344. 38186322 PMC10781419

[odi70044-bib-0043] Zondervan, K. T. , C. M. Becker , K. Koga , S. A. Missmer , R. N. Taylor , and P. Viganò . 2018. “Endometriosis.” Nature Reviews Disease Primers 4, no. 1: 9. 10.1038/s41572-018-0008-5. 30026507

