# Supplementary material for: Endometriosis‐Associated Periodontal Disease: A Large Cohort Perspective Study
Source: Oral Dis. 2025 Jul 26;32(1):201–15. doi: 10.1111/odi.70044 (PMC13031406; doi:10.1111/odi.70044)
Supplement: Supplementary file 1 — Figure S1. Weights assigned to responses based on the first dimension of multiple correspondence analysis. [file ODI-32-201-s001.docx]

(Q15B was excluded because it had a p-value > 0.25. Positive values are associated with a greater perception of having periodontal disease)

**Figure 1S.** Weights assigned to responses based on the first dimension of multiple correspondence analysis.
